# Exploration of potential molecular mechanisms and genotoxicity of anti-cancer drugs using next generation knowledge discovery methods

**DOI:** 10.12669/pjms.39.4.7427

**Published:** 2023

**Authors:** Peter Natesan Pushparaj, Mahmood Rasool, Muhammad Imran Naseer, Kalamegam Gauthaman

**Affiliations:** 1 Peter Natesan Pushparaj, PhD Associate Professor Center of Excellence in Genomic Medicine Research, Department of Medical Laboratory Technology, Faculty of Applied Medical Sciences King Abdulaziz University, Jeddah, Saudi Arabia; 2 Mahmood Rasool, PhD Professor Center of Excellence in Genomic Medicine Research, Department of Medical Laboratory Technology, Faculty of Applied Medical Sciences King Abdulaziz University, Jeddah, Saudi Arabia; 3 Muhammad Imran Naseer, PhD Professor Center of Excellence in Genomic Medicine Research, Department of Medical Laboratory Technology, Faculty of Applied Medical Sciences King Abdulaziz University, Jeddah, Saudi Arabia; 4 Kalamegam Gauthaman, MBBS, PhD Professor, Center for Transdisciplinary Research, Department of Pharmacology, Saveetha Dental, College and Hospitals, Saveetha Institute of Medical and Technical Sciences, Chennai, India

**Keywords:** Toxicogenomics, Anti-Cancer Agents, Cell Death, Apoptosis, Ingenuity Pathway Analysis, SwissTargetPrediction, WebGestalt

## Abstract

**Background & Objectives:**

Accurate identification of molecular and toxicological functions of potential drug candidates is crucial for drug discovery and development. This may aid in the evaluation of the risks of genotoxicity and carcinogenesis. In addition, in silico characterization of existing and new drugs might offer clues for future investigations and aid in the development of anticancer treatments. Using next-generation knowledge discovery (NGKD) methodology, we endeavored to establish a risk assessment of anticancer drugs for their molecular mechanism(s) and genotoxicity.

**Methods:**

This study was performed at the Faculty of Applied Medical Sciences, King Abdulaziz University (KAU), Jeddah, Saudi Arabia, in November 2022. Using innovative in silico model systems, we assessed the molecular mechanism of action and toxicity of around 20 distinct substances such as Deguelin, Etoposide, Camptothecin, Cytarabine (Ara-C), Cisplatin, Hydroxyurea, Trichostain A, Antimycin, Colchicine, 2-deoxyglucose, Tunicamycin, Thapsigargin, Vinblastin, Docetaxel, Oxaliplatin, Methotrexate, 5-flurouracil, Bleomycin, Taxol (Paclitaxel), and Apicidin. Using the Ingenuity Pathway Analysis (IPA) knowledge base, the number of targets for each compound was determined in silico. Subsequently, they were examined using Fisher’s exact test and Benjamini Hochberg Multiple Testing Correction (P<0.05) and submitted to core analysis with IPA to decode the biological and toxicological activities differently controlled by these drugs. In addition, a multiple comparison module in IPA was used to compare the core analyses of each molecule. In addition, we obtained the top 100 protein targets of Etoposide, Camptothecin, and Ara-C using SwissTargetPrediction, as well as the key pathways and gene ontologies affected by these drugs and disease associations using the WebGestalt tool.

**Results:**

We identified distinct toxicological signatures and canonical signaling pathways in tumor cell lines regulated by these 20 anticancer drugs. These signaling pathways included cell death and apoptosis in addition to molecular processes, p53 signaling, and aryl hydrocarbon receptor signaling. The TP53 signaling pathway is utilized by these agents to effectively trigger cell death and apoptosis, and p53 functions as a master regulator in a variety of cellular stress responses, including genotoxic stress.

**Conclusion:**

Our research has laid the groundwork for the discovery of additional biomarkers that assess both the safety and effectiveness of treatment. Our mechanism based “NGKD” tools have more relevance for the identification of safer therapies and has the potential to lead to the rational screening of drug candidates targeting specific molecular networks and canonical pathways implicated in cancer and genotoxicity. In addition, the combination of protein, microRNA and metabolome profiles may be essential for the development of translatable biomarkers for the safety and efficacy of pharmacotherapeutic agents.

Our research has laid the groundwork for the discovery of additional biomarkers that assess both the safety and the effectiveness of a treatment.

## INTRODUCTION

In drug discovery and development, an accurate understanding of the molecular and toxicological activities of anti-cancer drugs is absolutely necessary.^1^ In addition to this, deciphering the toxicological functions of drugs may be of use in the evaluation of the potential for genotoxicity and carcinogenesis.^2^,^3^ The in-silico characterization of known as well as novel compounds has the potential to yield leads for the development of drugs for the treatment of cancer. In addition to this, it may be of assistance in the process of rational screening for new candidate medications that target canonical pathways as well as novel gene networks that target the main Hall Marks of Cancer.^4^

To decode the mechanistic interpretation that was based on toxicological functions, molecular signaling pathways, and intracellular signaling molecules that were differentially impacted by 20 different anti-cancer drugs including Etoposide, Camptothecin, and Cytarabine (Ara-C), the next generation knowledge discovery (NGKD) approach was utilized in the current study.^5^,^6^ The NGKD approach utilizes the knowledge discovery databases such as Ingenuity Pathway Software to obtain the gene networks, canonical pathways, and upstream regulators regulated by a specific drug or natural product.

Besides, an array of open source in silico tools such as SwissTargetPrediction, and WebGestalt are available for further in-depth knowledge discovery.^7^-^11^ We aimed to establish the risk assessment of various drug candidates for genotoxicity and carcinogenesis using an approach known as NGKD. This was done to make the process of risk assessment of genotoxicity and carcinogenesis more straightforward. Our methodology has the potential to comprehensively assess a variety of genotoxic and non-genotoxic damage responses exhibited by both known and novel drug candidates.

## METHODS

This study was performed at the Faculty of Applied Medical Sciences, King Abdulaziz University (KAU), Jeddah, Saudi Arabia, in November 2022. This research did not require Institutional Review Board (IRB) clearance since neither animal models nor human volunteers were employed. We used the Ingenuity Pathway Analysis (IPA) software for knowledge discovery, data analysis, and interpretation.^6^

### In Silico Analysis using Ingenuity Pathway Analysis:

We used cutting-edge in silico model systems to assess the effectiveness and toxicity of over 20 distinct anti-cancer agents such as Deguelin, Etoposide, Camptothecin, Ara-C, Cisplatin, Hydroxyurea, Trichostain A, Antimycin, Colchicine, 2-deoxyglucose, Tunicamycin, Thapsigarin,Vinblastin, Docetaxel, Oxaliplatin, Methotrexate, 5-flurouracil, Bleomycin, Taxol (Paclitaxel), and Apicidin. To estimate the number of genes regulated by each drug in tumor cell lines, an in-silico analysis was performed using the Ingenuity Pathway Analysis (IPA) knowledge base (Qiagen, USA). They were then submitted to core analysis utilizing IPA to interpret both biological and toxicological functions differentially regulated by these drugs, using Fisher’s exact test and Benjamini Hochberg Multiple Testing Correction (P < 0.05).^7^-^9^

### Swiss Target Prediction:

To determine the molecular function and genotoxicity of Etoposide, Camptothecin, and Ara-C, target prediction was carried out using the SwissTargetPrediction online application, using updated bioactivity data and retrained and redefined similarity criteria.^7^,^9^,^10^ On the basis of the similarity between the query molecule and the curated collection utilizing 2D and 3D similarity measures inside the wider bioactivity data of ChEMBL version 23, ligand-based target prediction was done.^9^ A score greater than 0.5 suggests that the compounds have the same protein target.^9^ In reverse screening, the total score is used to determine the likelihood of targeting a certain protein. Dual-based reverse screening has proven effective for predicting macromolecular targets.^8^,^9^

### WebGestalt Analysis:

The WebGestalt tool (wGSEA) used the Over Representation Analysis (ORA) of the molecular targets of Etoposide, Camptothecin, and Ara-C generated from Swiss Target Prediction.^7^,^11^,^12^ Gene lists derived from large-scale -omics investigations were classified based on their biological, molecular, and cellular activities using wGSEA. wGSEA is a publicly accessible open-source platform that facilitates a more comprehensive, efficient, adaptable, and interactive functional enrichment analysis.^12^ The most recent version of wGSEA recognizes 155175 functional categories, 342 gene IDs, and 12 species, in addition to a large number of user-defined functional databases.^12^

## RESULTS

In this study, we investigated the efficacy and toxicity of 20 different anticancer compounds using state-of-the-art in silico methodologies. We used the IPA knowledgebase to obtain molecular targets in mammalian cells and tissues to study the mechanisms of action of 20 different compounds that are important in experimental therapeutics and molecular toxicology. This allowed us to study the mechanisms of action and genotoxicity of the compounds. Here, we compared each of the core analysis results using the multiple comparison module in IPA.

**Fig. 1:**
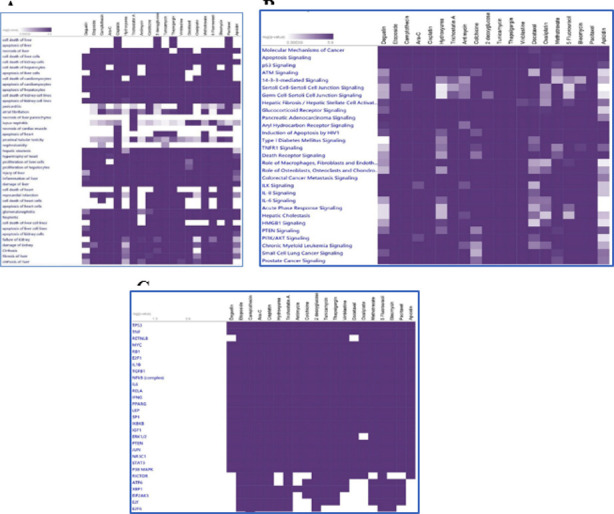
(A) Identification of unique toxicological effects of Etoposide, Cytarabine (Ara-C), Camptothecin, and other drugs (B) Impact on the Apoptosis Signaling, P53 Signaling, Aryl Hydrocarbon Receptor Signaling, and Molecular Mechanisms of Cancer, etc. in tumor cell lines. (C) Regulation of intracellular signaling molecules such as TP53, TNF, IL-1 beta, TGF-beta, NFkB complex, etc., to induce cell death and apoptosis of tumor cell lines.

This allowed the generation of hierarchical clusters for the most important canonical pathways, diseases, and biological and toxicological functions. Etoposide, Camptothecin, and Ara-C each have their own distinct set of toxicological effects, which we have categorized as hepatotoxicity, cardiotoxicity, and nephrotoxicity, respectively (Fig.1A). On the other hand, the majority of the compounds significantly regulated the molecular mechanisms of cancer in mammalian systems, including P53 Signaling, Apoptosis Signaling, and Aryl Hydrocarbon Receptor Signaling (Fig.1B), and utilized the TP53 signaling pathway to effectively induce cell death and apoptosis in tumor cell lines (Fig.1C).

The tumor suppressor p53 acts as a key regulator of a wide variety of cellular stress responses, including genotoxic stress (Fig.1C). Using the SwissTargetPrediction program, we were able to extract the top100 protein targets for etoposide, camptothecin, and Ara-C. We used WebGestalt analysis for pathways, gene ontology, and disease correlations for etoposide targets (Fig.1D), camptothecin targets (Fig.1E), and Ara-C targets (Fig.1F).WebGestalt analysis of etoposide protein targets using the GLAD4U disease database showed that gene sets implicated in Drug Toxicity, Ischemia, Head and Neck, Neoplasms, Drug interaction with drug, Disease Progression, Myocardial Ischemia, Vascular Diseases, Inflammation, overall survival, and Carcinoma were significantly (P<0.05) impacted by the treatment (Table-I).

**Table-I:** WebGestalt analysis of etoposide protein targets using GLAD4U disease database.

Gene Set	Description	Size	Expect	Ratio	P Value	FDR
PA443937	Drug Toxicity	191	0.88859	20.257	<2.2e-16	<2.2e-16
PA444645	Ischemia	221	1.0282	16.534	2.2204e-16	1.5093e-13
PA444352	Head and Neck Neoplasms	387	1.8004	11.664	<2.2e-16	<2.2e-16
PA165108622	Drug interaction with drug	494	2.2982	11.313	<2.2e-16	<2.2e-16
PA446728	Disease Progression	333	1.5492	10.973	2.1716e-13	7.3807e-11
PA446459	Myocardial Ischemia	361	1.6795	10.122	7.9903e-13	2.4140e-10
PA446021	Vascular Diseases	491	2.2843	8.3177	1.0061e-12	2.7355e-10
PA444620	Inflammation	565	2.6286	7.9892	1.1779e-13	5.3381e-11
PA166123207	overall survival	648	3.0147	7.2976	1.7386e-13	6.7533e-11
PA443610	Carcinoma	785	3.6521	6.5716	1.1036e-13	5.3381e-11

Using the GLAD4U disease database, the WebGestalt analysis of camptothecin protein targets found that gene sets implicated in Drug Interaction with Drug, Vascular Diseases, Inhibitory Concentration 50, Drug Resistance, Pathologic Processes, Cardiovascular Diseases, Depression, Pain, Breast Diseases, and Mouth Neoplasms were significantly (P<0.05) impacted (Table-II). WebGestalt analysis of Ara-C protein targets using the GLAD4U disease database showed that gene sets implicated in Drug Interaction with Drug, Inhibitory Concentration 50, Drug Resistance, Overall survival, Lung Neoplasms, Drug Toxicity, Neoplasms, Hormone-Dependent Colorectal Neoplasms, Neoplasms, and Mouth Neoplasms were significantly (P<0.05) impacted by the cytotoxic effects of the drug (Table-III).

**Table-II:** WebGestalt analysis of camptothecin protein targets using GLAD4U disease database.

Gene Set	Description	Size	Expect	Ratio	P Value	FDR
PA165108622	Drug interaction with drug	494	2.4205	11.155	<2.2e-16	<2.2e-16
PA446021	Vascular Diseases	491	2.4058	8.7289	2.2871e-14	3.1093e-11
PA166159283	inhibitory concentration 50	74	0.36258	30.338	7.1498e-14	6.4801e-11
PA165111675	Drug Resistance	334	1.6365	10.388	5.5445e-13	3.7688e-10
PA445265	Pathologic Processes	747	3.6601	6.2839	1.1116e-12	6.0446e-10
PA443635	Cardiovascular Diseases	583	2.8566	7.0014	5.9479e-12	2.6954e-9
PA447278	Depression	252	1.2347	11.338	2.2748e-11	8.8359e-9
PA445208	Pain	310	1.5189	9.8754	2.9616e-11	1.0066e-8
PA443559	Breast Diseases	455	2.2294	7.6254	7.4161e-11	2.2405e-8
PA444972	Mouth Neoplasms	182	0.89176	13.457	9.2674e-11	2.5198e-8

**Table-III:** WebGestalt analysis of Ara-C protein targets using GLAD4U disease database.

Gene Set	Description	Size	Expect	Ratio	P Value	FDR
PA165108622	Drug interaction with drug	494	2.3716	7.5898	2.0261e-11	5.5091e-8
PA166159283	inhibitory concentration 50	74	0.35526	25.334	7.9251e-11	7.1828e-8
PA165111675	Drug Resistance	334	1.6035	9.3547	6.2592e-11	7.1828e-8
PA166123207	overall survival	648	3.1109	6.1075	2.1463e-10	1.4590e-7
PA444818	Lung Neoplasms	418	2.0067	7.4748	1.3978e-9	7.6012e-7
PA443937	Drug Toxicity	191	0.91695	11.996	1.9794e-9	8.9699e-7
PA166048737	Hormone-Dependent Neoplasms	196	0.94096	10.627	3.5467e-8	0.000013776
PA446108	Colorectal Neoplasms	388	1.8627	6.9791	4.2900e-8	0.000014581
PA445062	Neoplasms	1217	5.8426	3.7655	5.2846e-8	0.000015965
PA444972	Mouth Neoplasms	182	0.87374	10.300	2.3162e-7	0.000062978

## DISCUSSION

The accurate molecular and toxicological identification of potential drugs and natural products is necessary for the research and development of new pharmaceuticals.^1^ Using the NGKD methodology, we conducted this study to investigate the molecular processes and disease associations of twenty different anti-cancer agents. We deciphered that the anticancer compounds such as etoposide, camptothecin, and Ara-C had an effect on the toxicological consequences such as hepatotoxicity, cardiotoxicity, and nephrotoxicity. They are responsible for the regulation of the Molecular Mechanisms of Cancer, as well as the Signaling of Apoptosis, P53, and Aryl Hydrocarbon Receptors.

In addition, these chemicals utilize the TP53 signaling pathway to effectively induce cell death and apoptosis in tumor cell lines. In many different cellular stress responses, including the response to genotoxic stress, the tumor suppressor p53 acts as a major regulator, and mutations in the p53 gene result in cancer.^13^,^14^ We found that p53 is a significant factor in the transcriptome changes generated by genotoxicity at the gene level. Topoisomerase expression has been found to be increased in a variety of solid tumors and the inhibition of topoisomerases is one of the key strategies in the development of anti-cancer therapy.^15^ Etoposide is a semisynthetic product of podophyllotoxin, which is obtained from Podophyllum peltatum, also known as mandrake root.^16^,^17^

It binds to topoisomerase II and inhibits its activity, resulting in breaks in either single or double strands of DNA, as well as obstruction of DNA replication and transcription, leading to apoptotic cell death. It mostly affects the cell cycle’s G2 and S stages.^16^ Our NGKD approach also showed that etoposide can interact with proteins involved in drug toxicity and capable of causing liver inflammation and cirrhosis.^18^ Moreover, it was associated with the different neoplasms, myocardial ischemia, etc. Carcinogenic effects of etoposide was also observed in experimental animals^19^ and the induction of secondary malignancies.^20^ There is also evidence that chromosomal translocations can be triggered by topoisomerase II-mediated DNA strand breaks induced by etoposide and other drugs.

These translocations may lead to some forms of leukemia.^19^ Camptothecin is an alkaloid that was originally extracted from the stem wood of the Camptotheca acuminata tree, which is native to China.^22^ Antitumor action has been found in a few different semisynthetic analogs of the compound camptothecin. During the S phase of the cell cycle, it preferentially stabilizes topoisomerase I-DNA covalent complexes, which limits replication of single-strand DNA breaks and causes the DNA replication machinery to encounter potentially lethal double-strand breaks.^23^ Our NGKD approach showed that camptothecin can interact with proteins implicated in cardiovascular diseases, neoplasms, drug resistance, pathological process, breast diseases, and mouth neoplasms. Ara-C, an antineoplastic agent and cytosine analogue, is often used in the treatment of leukemia, particularly acute non-lymphoblastic leukemia.^24^

Additionally, it inhibits viral replication and suppresses the immune system. Within the cell, Ara-C is converted to the triphosphate form, where it competes with cytidine for DNA inclusion. Due to steric hindrance by the arabinose sugar, DNA replication is halted, particularly during the S phase of the cell cycle. Similarly, this drug inhibits DNA polymerase, leading to a reduction in DNA replication and repair. Our NGKD approach showed thatAra-C can interact with proteins implicated in a variety of neoplasms, drug interactions, and drug toxicity.

Ara-C may cause transitory increases in blood enzyme and bilirubin levels and that this may play a role in the development of clinically evident acute liver damage with jaundice.^25^ It was recently reported that Ara-C caused bradycardia in a patient receiving it along with idarubicin.^24^ Hence, it is essential to point out that our mechanism based “NGKD” tools have more relevance for the identification of safer therapies and has the potential to lead to the rational screening of drug candidates targeting specific molecular networks and canonical pathways implicated in cancer and genotoxicity.

**Fig.2:**
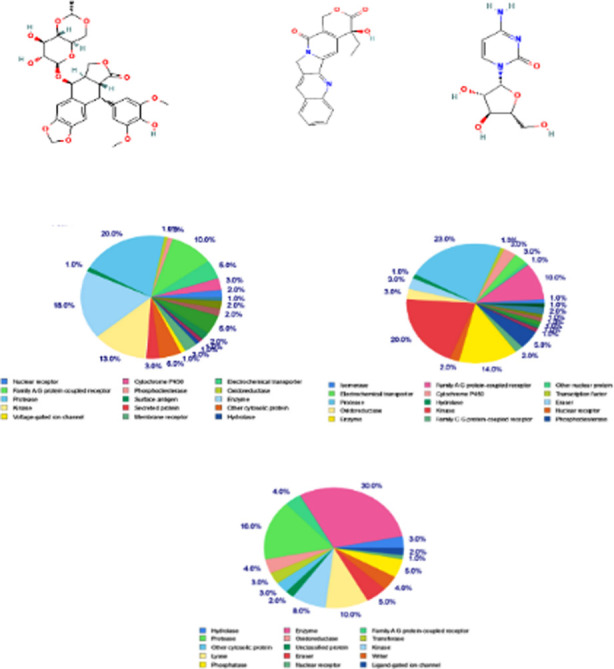
The query molecules used for SwissTargetPrediction analysis to decipher key protein targets. (A) Etoposide (PubChem CID: 36462) (B) Camptothecin (PubChem CID: 24360) (C) Cytarabine (Ara-C) (PubChem CID: 6253) (D) Etoposide Targets (E) Camptothecin Targets (F) Cytarabine Targets.

### Limitations:

We conducted this study using NGKD methods based on in silico tools. Hence, it is important to validate these findings using appropriate in vitro and in vivo studies using compounds with anti-cancer potential. In addition, a combination of high-throughput proteomics, transcriptomics, and metabolomics experiments may be necessary to decipher translatable biomarkers for the safety and effectiveness of new pharmacotherapeutic drugs.

## CONCLUSIONS

Using a variety of “NGKD” platforms, we were able to identify the processes underlying the genotoxicity of many different anti-cancer medicines. The advantage of our study is that the “NGKD -based approach” makes the mechanism-based risk estimation for the compounds with anti-cancer potential easy and precise since there are no broad-mechanism-based assays and the methods that are currently available are laborious and time-consuming. It is important to note that our mechanism based “NGKD” approach may be considered as a preliminary step in the development of new treatments and may be adopted for rational screening of novel drugs with anti-cancer potential in the future.

### Author Contribution:

**PNP, MR, MIN, KG:** Conceptualization, methodology, data analysis, writing the original draft, co-responsibility and integrity of the study.

**MR, MIN:** Manuscript correction and editing.

**KG:** Manuscript final editing for submission.
